# Impact of living with bipolar patients: caregivers’ mental health and quality of life

**DOI:** 10.1192/j.eurpsy.2023.773

**Published:** 2023-07-19

**Authors:** M. Djelassi, H. Jemli, M. Ben Daly, R. Jomli

**Affiliations:** 1 Psychiatry deparment A, Razi Hospital, Manouba; 2 Faculty of medicine of Tunis, University Tunis Elmanar, Tunis, Tunisia

## Abstract

**Introduction:**

Bipolar disorder is a common chronic illness with progressive intermittent. The new health policy, which advocates the deinstitutionalization of patient’s mental illnesses, caused, for a part, a transfer of the load of these patients from the specialized care services to natural caregivers, which can involve a great burden for family members as well as an altered quality of life, anxiety and depressive symptoms.

**Objectives:**

The aim of the study was to evaluate the rate of anxiety, depression and the repercussions on life quality in natural caregivers of patients with bipolar disorder.

**Methods:**

This is a descriptive cross-sectional study that involved a group of 50 caregivers of patients with bipolar disorder treated at psychiatry department ‘A’ at Razi Hospital. We applied a questionnaire recording the different socio-demographic data. To determine the impact on the caregivers mental health and life quality we used the WHOQOL-brief (World Health Organization Quality Of Life-abbreviated version) and the HAD (Hospital Anxiety and Depression) Scale.

**Results:**

Our sample consisted of 32 women and 18 men. The mean age was 52.12 years with extremes ranging from 28 to 79 years. A majority were parents (60%), 18% spouses, 16% siblings and 6% descendants.

The repercussions of management of patients with bipolar disorder on the life and health of the caregivers were significant. Indeed, more than half of the caregivers (52%) (n=26) had a definite anxiety symptomatology on the Hospital Anxiety and Depression Scale (HAD). Twenty-two percent (n=11) presented definite depressive symptomatology and thirty percent (n=15) doubtful depressive symptomatology on this same scale. The most impaired domains on the World Health Organization Quality of Life Scale-version abbreviated (WHOQOL) were first: the environment domain with an average of 25.9 and second: the physical health domain with an average of 23.9.

**Conclusions:**

Being a caregiver for a patient with bipolar disorder is associated with a great burden that can be the cause of anxiety-depressive complications and an alteration in the caregiver’s quality of life. It is important to assess this burden and its repercussions in order to preserve good family dynamics and ensure the proper functioning of the helping relationship and consequently improve the prognosis.

**Disclosure of Interest:**

None Declared

